# Food Neophobia in Brazilian Children: A Nationwide Cross-Sectional Study Comparing Neurodivergent and Neurotypical Children with and Without Dietary Restrictions

**DOI:** 10.3390/nu17081327

**Published:** 2025-04-11

**Authors:** Priscila Claudino De Almeida, Nathália França Freire, Letícia Leal de Oliveira, Eduardo Yoshio Nakano, Ivana Aragão Lira Vasconcelos, Renata Puppin Zandonadi, Raquel Braz Assunção Botelho

**Affiliations:** 1 Postgraduate Program in Human Nutrition, University of Brasília, Brasília 70910-900, Brazil; nprialmeida@gmail.com; 2 University of Brasília, Brasília 70910-900, Brazil; 3 Department of Statistics, University of Brasília, Brasília 70910-900, Brazil; 4 Department of Nutrition, University of Brasília, Brasília 70910-900, Brazil; renatapz@unb.br (R.P.Z.); raquelbotelho@unb.br (R.B.A.B.)

**Keywords:** food neophobia, neurodiversity, food restriction, child, caregiver perception

## Abstract

**Objective:** This study aims to compare food neophobia (FN) in groups of neurodivergent and neurotypical children with and without food restrictions. **Methods:** This cross-sectional study used a convenience sample of participants from all Brazilian Federative Units through the snowball method. Participants were separated into four groups for comparison: neurodivergent children without dietary restrictions; neurodivergent children with dietary restrictions; neurotypical children with dietary restrictions; and neurotypical children without dietary restrictions. The Brazilian Children’s Food Neophobia Questionnaire (BCFNeo) was selected and administered to caregivers of Brazilian children between four and eleven years old. The categorization of FN scores followed a previously validated protocol. **Results:** The sample was composed of the caregivers of 2387 children. Considering the sample as a whole (neurodiversity), the prevalence of high FN was 44.2%. Boys are significantly more neophobic (*p* < 0.001) than girls. FN does not decrease with age. Older children (eight to eleven years) are equally neophobic as younger children (four to seven years). The prevalence of high FN was greater in neurodivergent children (without dietary restrictions—62.8% and with dietary restrictions—62.4%) than in neurotypical children (with dietary restrictions—29.4% and without dietary restrictions—33.4%). **Conclusions:** FN is more prevalent in boys and neurodivergent children, regardless of food restrictions and age. More longitudinal and experimental studies are necessary to evaluate the factors influencing FN in these groups and to develop intervention strategies.

## 1. Introduction

Neurodiversity expresses the diversity of cognitive and sensory functions in humans. This implies recognizing that there are clinically and psychologically healthy individuals with a normative pattern of neurodevelopment, known as neurotypicals, and individuals whose cognitive and/or sensory functioning differs from the neurotypical population, referred to as neuroatypical or neurodivergent [1], such as people with autism spectrum disorder (ASD), Asperger’s syndrome, attention deficit hyperactivity disorder (ADH), Alzheimer’s disease, bipolar disorder, depression, DiGeorge syndrome, Down syndrome, dyscalculia (difficulty with math), dysgraphia (difficulty with writing), dyslexia (difficulty with reading), dyspraxia (difficulty with coordination), epilepsy, intellectual disabilities, obsessive–compulsive disorder, Prader–Willi syndrome, sensory processing disorders, social anxiety (a specific type of anxiety disorder), Tourette’s syndrome, Williams syndrome, and any number of other psychiatric, mental and neurological classifications [1,2,3]. Several neurodivergent conditions, such as ADHD and ASD, in the evolutionary sense of humanity, should not be treated as disorders. Neurodivergence has favorable variations that add value to both the individual and the group [4].

Each person’s brain develops uniquely. Neurodivergence is not preventable or curable and results in different strengths and challenges [3]. Therefore, it should not be analyzed as a list of deficits and dysfunctions but rather as natural cognitive variations, celebrating diversity with distinct aspects that contribute to the evolution of technology and culture [1]. The wide variety of conditions associated with neurodivergence, along with the high worldwide prevalence of neurodivergence, estimated to be between 15% and 20%, reinforces the need for targeted public policies [5].

Neuroatypical children tend to have a higher prevalence of feeding problems than children with neurotypical development. Eating difficulties in childhood are still challenging for child development health professionals, and there is no consensus on the definition and conceptual structure of eating difficulties. This challenges the description of the multiple factors involved and increases the difficulty of the identification of appropriate interventions [6]. Children with special needs, some disabilities, or medical conditions who are reported as selective eaters may have food neophobia (FN) [7], defined as the reluctance to eat or the avoidance of eating unfamiliar or novel foods [8,9,10,11]. Neurodivergent people present a full range of disordered eating behaviors [12,13]. However, much research focuses on evaluating eating behaviors and diet quality in individuals with ASD [12,14,15,16,17,18]. FN affects people with ASD [19,20], Down syndrome [21], and Tourette’s syndrome [22], among other conditions.

Another particularity that may be more prevalent in neurodivergent people is dietary restrictions. Children with Down syndrome are more likely to develop intolerances to certain foods and celiac disease [23]. ASD is a risk factor for food allergy, and vice versa [24]. Adverse reactions to foods include food allergies, sensitivities, and intolerances to a food or to a nutrient/ingredient that composes the food [25], which demonstrate increasing incidence in the worldwide population [26]. Food intolerance is estimated to affect up to 20% of the worldwide population [27], and about 1% of adults and 4% of children worldwide have a food allergy [28].

Considering the expected feeding difficulties in neurodivergent children and the cases in which early dietary restrictions are necessary for both neurotypical and neurodivergent children, our study hypothesizes that a significant number of Brazilian neurodivergent children and those with dietary restrictions will exhibit high levels of FN, with no differences based on sex or age when compared to neurotypical children or those without dietary restrictions.

Despite dietary restrictions, individuals must maintain a balanced diet with an adequate nutritional profile to compensate for the foods they avoid [26]. Access to nutritious and healthy food is one of the most important aspects of good health, but it can be affected by several factors, including food choices and acceptance [29]. Food-adequate choices can promote a nutritionally balanced, tasty, and culturally appropriate diet that benefits the individual and the planet, but these choices can be prejudiced by FN [30]. FN results in low nutritional quality in the diet and can create a risk of malnutrition and nutritional deficiencies due to the lack of several important foods. This does not always result in inadequate BMI but can influence weight and development [31]. Neurodivergent people experience poorer treatment outcomes, and research on these individuals is scarce, especially considering neurodiversity and its relation to FN [13]. Since FN can impair or be impaired by several factors, such as food restrictions and neurodivergence, this study aims to compare FN in groups of neurodivergent and neurotypical children with and without food restrictions.

## 2. Materials and Methods

This descriptive cross-sectional study was conducted in Brazil using a convenience sample of caregivers of Brazilian children with dietary restrictions and neurodivergence. Following the Declaration of Helsinki guidelines [32] and Resolution No. 466 of 12 December 2012 [33], the Health Sciences Ethics Committee of the University of Brasilia analyzed and approved this study (No. 5.438.498).

### 2.1. Participants

This study recruited a convenience sample of participants from all Federative Units of Brazil using the snowball method. This method is helpful for obtaining a larger sample at a low cost from people with specific conditions, but it tends to be more homogeneous in terms of social, cultural, and demographic characteristics. Therefore, the sample may not adequately reflect the diversity of the Brazilian population [34].

Participants needed to know their children’s eating habits and behaviors and provide consent to participate in the research to be included in this study. Participants indicated in a specific questionnaire topic whether the children had been diagnosed by a doctor with any medical condition or disease and identified those who were diagnosed with food-related disorders requiring dietary restrictions. Based on the reports of conditions and diagnoses, the researchers grouped the neurodivergent children, separating them from the neurotypical children, as well as those with and without dietary restrictions. This study considered dietary restrictions for children who restricted food intake because of food-related disorders such as allergies, intolerances, and sensitivities [25]. These included diseases or medical conditions that, as part of their treatment, require the exclusion of certain foods or nutrients, such as diabetes, celiac disease, enzyme deficiencies (such as lactose intolerance), and others.

The sample was separated into four groups for comparison: (G1) neurodivergent children without dietary restrictions; (G2) neurodivergent children with dietary restrictions; (G3) neurotypical children with dietary restrictions; and (G4) neurotypical children without dietary restrictions. Considering an effect size of 0.4 (moderate effect size) when comparing pairs of groups, a 5% significance level, and a power of 80%, the sample size calculation indicates a minimum of 99 participants per group, resulting in a total minimum sample of 396 participants.

### 2.2. Instruments and Application

Psychometric validation of self-regulation instruments in food behavior studies has been a crucial factor for reliable assessment [35]. The Brazilian Children’s Food Neophobia Questionnaire (BCFNeo) was selected since it is a specific instrument developed and validated in Brazil to identify FN in Brazilian children as reported by their caregivers [8]. In creating the Brazilian instrument, the studies by Pliner and Hobden [9], Damsbo-Svendsen and collaborators [36], Hollar and collaborators [37], and Allirot and collaborators [38] were used as a basis, in addition to the authors’ expertise in creating new questions based on a review of the topic [39]. The Delphi method was used for the instrument’s content validation and semantic evaluation [8]. For this research, the instrument, composed of sociodemographic data and BCFNeo [8], was available through the online platform Google Forms^®^ and widely disseminated on social networks (such as Instagram^®^ [40], Facebook^®^ [41], Twitter^®^, and X^®^) and via messaging apps and email. The data collection process occurred from November 2022 to December 2024.

The first part of the instrument collected data on sociodemographic aspects: the caregiver’s profile (age, sex, degree of kinship, marital status, family income, and educational level) (Supplementary File—Table S1) and, finally, the child’s profile (nationality, sex, age—Table 1 and diagnoses—Table S2). The instrument was self-administered and completed by caregivers. Caregivers were instructed that only one of them should respond to the instrument. Those with more than one child in the research age group could fill in the questionnaire as often as necessary, according to the number of children.

The categorization of the FN score followed a previously validated protocol [11]. The items were divided into three domains: neophobia in general (FNgen), neophobia for fruits (FNfru), and neophobia for vegetables (FNveg). Each domain contained a similar number of items, allowing for a better analysis of the scores for the entire instrument (25 items) and for each domain. This item distribution allowed for the assessment of neophobic traits in each domain separately or through the complete instrument [11].

The answers were inverted and coded to assume values from 0 to 4 for each question. The general domain score (with 9 items) ranges from 0 to 36; the score for the fruit domain (8 items) ranges from 0 to 32; the score for the vegetable domain (8 items) ranges from 0 to 32; and the total score (25 items) ranges from 0 to 100 (9). The categorization of FN was defined according to scores: (i) up to 40 points: low neophobia, (ii) from 41 to 65 points: moderate neophobia, and (iii) 66 points or more: high neophobia. Therefore, for each domain, a low FN is up to 13 points, moderate FN is from 14 to 21 points, and high FN is from 22 points and above [11].

Questionnaires in which participants stated they did not want to participate in this study or answered the section on health characteristics incompletely were excluded. In order to classify FN, questions on this topic were marked as mandatory, but caregivers could stop answering and withdraw from participation at any time, as described.

### 2.3. Statistical Analysis

Descriptive statistics were presented as means and standard deviations or frequencies and percentages. The analysis considered two age groups (4–7 and 8–11 y/o), sex (female and male), and diagnostic categories (four groups). The neophobia scores were compared between the sexes and ages of the children using an independent Student’s *t*-test and among the diagnostic categories using one-way ANOVA with Tukey’s post hoc test. These scores were also categorized according to three ordinal levels. The Mann–Whitney U test was used to compare neophobia levels between sexes and the child’s age. Comparisons of FN levels among diagnoses were performed using the Kruskal–Wallis test, followed by Dunn’s post hoc test. The normality of the observations was tested using the Kolmogorov–Smirnov test with Lilliefors correction. All tests considered two-tailed hypotheses and a significance level of *p* < 0.05. After extraction from the Google Forms^®^ platform (https://docs.google.com/forms/u/0/, accessed on 31 March 2025), analysis was performed using SPSS^®^ 20.0 software and Excel^®^.

## 3. Results

**Figure 1:**
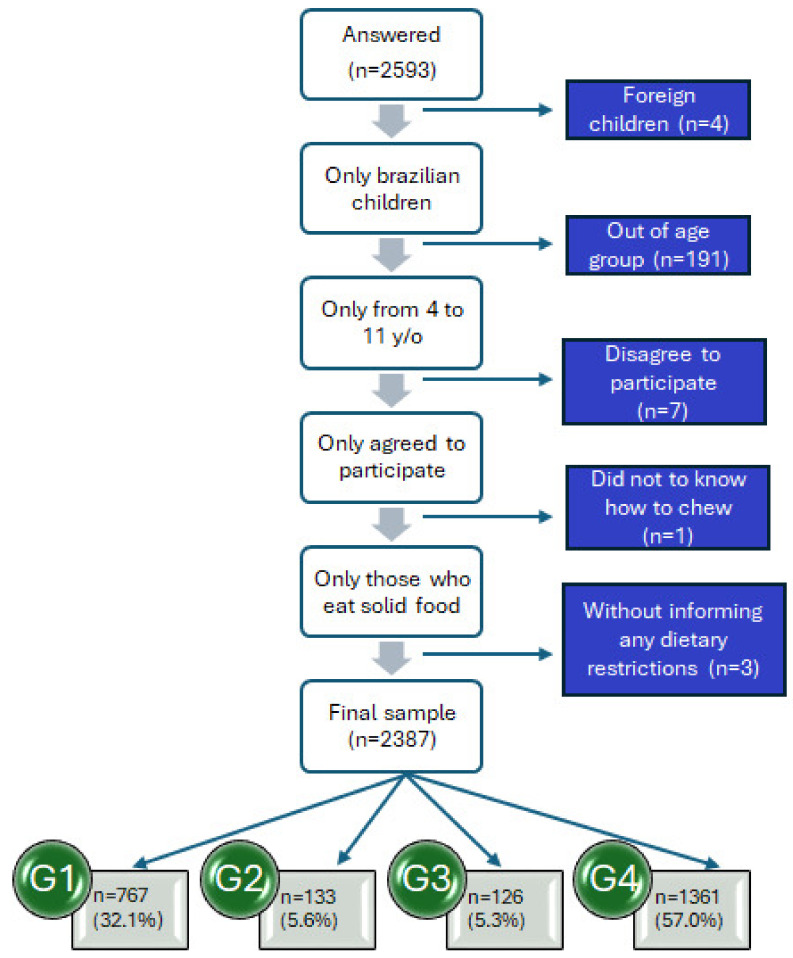
Schematic representation of the study sample (Brazil 2022–2024). G1: neurodivergent children without dietary restrictions; G2: neurodivergent children with dietary restrictions; G3: neurotypical children with dietary restrictions; and G4: neurotypical children without dietary restrictions.

According to Figure 1, of the 2593 participants who accessed the questionnaire, 2361 constituted the final sample. The same figure shows the reasons for exclusion and the number of participants excluded from the sample. The sample was stratified into four groups (G1, G2, G3, G4) to compare FN levels based on neurodivergence diagnosis and dietary restrictions.

Children of all ages (4 to 11 years) participated, with the majority being boys (n = 1378; 57.7%), although the sample was well-balanced. The group of neurotypical children without dietary restrictions (G4) had the most extensive distribution (n = 1361; 57.0%), as shown in Table 1. In the neurodivergent group, the majority were children with ASD (n = 604; 25.3%). Most of the dietary restriction groups had food intolerances (Table 1).

**Table 1:** Sociodemographic data and children profile (n = 2387).

	Categories	Sample	
		n	%
Gender	Female	1009	42.3
	Male	1378	57.7
Age	4 years old	460	19.3
	5 years old	406	17.0
	6 years old	321	13.4
	7 years old	243	10.2
	8 years old	272	11.4
	9 years old	276	11.6
	10 years old	221	9.3
	11 years old	188	7.9
Diagnose ^a^	Down’s syndrome	266	11.1
	Autism spectrum disorder	604	25.3
	Sensory processing disorder	13	0.5
	Attention deficit hyperactivity disorder (ADHD)	37	1.5
	Giftedness/high-ability	4	0.1
	Epilepsy	8	0.3
	Intellectual disability	3	0.1
	Heart disease	6	0.2
	Thyroid diseases ^b^	6	0.2
	Eating disorders ^c^	20	0.8
	Food intolerance ^d^	158	6.6
	Food allergies ^e^	136	5.7
	Celiac disease	4	0.1
	G6PD enzyme deficiency	2	0.1
	Diabetes	1	0.1
	Inflammatory bowel disease	1	0.1
	Colitis	1	0.1
	Gluten sensitivity	2	0.1
	Fructosemia	1	0.1

^a^ Children may have one or more diagnosis. ^b^ Hypothyroidism, hyperthyroidism, or Hashimoto’s thyroiditis. ^c^ Anorexia/bulimia. ^d^ Such as lactose intolerance, gluten intolerance, or dye intolerance. ^e^ Such as allergies to cow’s milk protein, fish, shrimp, or chocolate.

Considering the sample as a whole (neurodiversity), the prevalence of low and high FN was 24.1% and 44.2%, respectively (Table 2).

Boys had a greater distribution in high FN of BCFNeoTot (50.7%) compared to girls (35.4%) (Table 3). Boys were more food neophobic in all domains than girls. There were no differences between younger and older children.

Neurodivergent children with and without dietary restrictions had the highest means in BCFNeoTot compared to neurotypical children (Table 4). The prevalence of high FN was 62.8% in neurodivergent children without dietary restrictions, 62.4% in neurodivergent children with dietary restrictions, 29.4% in neurotypical children with dietary restrictions, and 33.4% in neurotypical children without dietary restrictions. Neurodivergent children with dietary restrictions were less food neophobic than those without dietary restrictions in all domains (FNgen, FNfru, FNveg) and in total (BCFNeoTot).

## 4. Discussion

This is the first study to measure FN in the context of neurodiversity and dietary restrictions related to food disorders. Other studies have been conducted with neurodivergent individuals, such as those with autism [19], children with Down syndrome [21], and mixed populations [11,42], but they did not compare the groups or evaluate dietary restrictions. Furthermore, this study has the largest sample of Brazilian children evaluated for FN, as an instrument for this population was only recently developed [8,11,19,42,43].

The first nationwide study in Brazil that measured and characterized FN involved caregivers of 1112 neurodiverse Brazilian children [11]. The prevalence of high FN was 33.4%. In our study, this same prevalence (33.4%) was observed only in the neurotypical children without dietary restrictions group. It is important to mention that this prevalence is much lower than that found in neurodiverse children with or without dietary restrictions (Table 4).

Food restrictions caused by allergies may increase levels of FN. This occurs because negative experiences with foods and anxiety about possible allergic reactions restrict food variety. Parents’ and caregivers’ fear of adverse reactions intensifies the fear of trying new foods, making the diets of allergic children even more restricted [44]. The elimination diet is recommended in cases of food-related disorders, and it is necessary to make appropriate substitutions. In these cases, the micro- and macronutrient content should not be the only factor considered; attention must also be given to food components, aiming for a balanced diet without nutritional risks. Natural, healthy foods should be prioritized over unhealthy, industrialized ones, as the quality of the diet can impact the immune system. Food diversity in children’s diets has been associated with the prevention of allergies. Therefore, one of the recommendations is that these children increase their food variety and prioritize the consumption of healthy foods [45].

Neurotypical children with dietary restrictions had the lowest prevalence of high FN, at 29.4%. One possible reason for this group having a lower level of FN compared to neurotypical children without restrictions (33.4%) is that those with dietary restrictions already need to avoid certain foods due to allergies, sensitivities, or intolerances. Thus, when they have the opportunity, they seek food alternatives to expand their dietary options. On the other hand, caregivers may be hesitant to allow children with allergies to try new foods, which could lead them to “normalize” and underestimate the children’s refusal to consume unfamiliar foods. More studies should be conducted to evaluate this hypothesis and explain the factors involved.

A study showed that Brazilian children with gluten-related disorders (GRD) who were also using BCFNeo had a prevalence of high FN of 29.2% [46], similar to our results for the group with dietary restrictions (29.4%). In the capital of Brazil, the Federal District (FD), a study involving 595 children of the same age found a high FN prevalence of 42.9%, which is slightly lower than in our study [42].

Compared to children without allergies, children with allergies demonstrated a lower ability to recognize sour, salty, and bitter flavors [47]. This point may potentially explain why neurotypical children with dietary restrictions have a lower distribution of high FN for fruits (24.6%) and vegetables (36.5%) compared to the general population (42.1%). This may be one potential reason why neurodivergent children with dietary restrictions have lower levels of high FN compared to neurodivergent children without dietary restrictions. Future studies are necessary to explore this topic.

Neurodivergent children with dietary restrictions had a similar high FN to neurodivergent children without dietary restrictions, at 62.4% and 62.8%, respectively. These values were expected to be high since a study conducted in Brazil [19] that included 593 autistic children between 4 and 11 years old showed a high prevalence of FN at 73.9%. A systematic review was conducted to gather clearer evidence on the relationship between food selectivity or food neophobia (FN) and autism spectrum disorder (ASD) in children up to 14 years of age, using nine clinical studies from 1966 to 2021. The results indicated that children with ASD are more neophobic than neurotypical children [48]. When caregivers pressure children with ASD to eat, FN may increase. This was one of the findings from a study conducted with caregivers of 160 Chinese children with autism aged 2 to 7 years. Caregivers who were most concerned about their children’s nutritional status were more likely to replicate these pressured behaviors during mealtimes. Another observation was that the greater the social deficits, the greater the neophobia [49]. Although our study did not exclusively include children with ASD in the neurodivergent group, they represent a considerable proportion (25.3%).

Compared to neurotypical children, neurodivergent children with ADHD, ASD, and Tourette’s syndrome have greater sensitivity, in general, to taste, smell, touch, and visual/auditory information. This sensory sensitivity restricts these children’s eating habits; therefore, their eating patterns may be similar [50]. This aspect should be highlighted since our neurodivergent sample includes children with ADHD, ASD, and sensory processing disorder.

The prevalence of high FN in children with Down syndrome is 41.1%. The only study that evaluated FN in this population used the BCFNeo with the same methodology [21]. A total of 231 Brazilian children aged 4 to 11 participated in the study. Of the participants, 72.7% had only DS, without any other medical condition [21]. The prevalence of high FN may be different from that found in our neurodivergent children because children with DS do not have sensory processing disorder, which increases FN [21,50].

Our results indicate that FN did not decrease with advancing age. Older children (8 to 11 years) are as food neophobic as younger children (4 to 7 years). The literature indicates that FN tends to decrease [51], but studies in Brazil involving children contradict this finding [11,19,42,46]. More studies should be conducted in the country to explain the factors involved.

Our study showed that boys were more neophobic (in BCFNeoTot and all domains), as shown in previous Brazilian studies [11,42,46]. The only study in Brazil conducted using a validated instrument that did not show differences between the sexes was conducted on children with ASD [19]. However, children with ASD are highly selective and neophobic due to conditions linked to autism, such as sensory processing disorder. More exploratory or qualitative studies are needed to determine the real reasons why Brazilian boys are more food neophobic.

It is essential to assess FN using validated instruments and appropriate methodologies. In this way, it is possible to measure FN, to identify the food group in which it is most prevalent, and to differentiate how it manifests itself in different environments [11]. It is necessary to identify the root causes of eating difficulties and the mechanisms involved [12]. Caregivers, health professionals, and educators, when dealing with neophobic children, should promote a calm, positive environment without distractions. Additionally, they should refrain from using food as a form of reward, blackmail, or pressure to encourage children to eat. It is important not to force children but to encourage them to interact with food and consume it. New foods should be introduced gradually using food and nutrition education strategies. Whenever possible, children should be encouraged to participate in interventions that involve them in all stages of the food process, including planting, caring for, touching, smelling, selecting, buying, preparing, cooking, setting the table, making a shopping list, trying, and consuming [21]. Professionals should not claim that FN is due to neurodivergence in order to blame children for their behavior. Although we know that neurodivergent children experience greater FN, they also have the right to treatment and interventions, and they should not be neglected.

Despite the potential limitations of using a convenience sample and snowball sampling (SS), other studies that evaluated FN in Brazil have followed this same methodology [11,19,42,46]. Brazil has a large territory; this method is simple, cheap, and practical [42]. It contributed to our large sample, the largest in the country, in studies that evaluated FN. This study’s conclusions should also be considered with caution, as the sample using this method, although large, may have lower representativeness, as it tends to be more homogeneous in terms of social, cultural, and demographic characteristics [34].

It should also be considered that there is a response bias in cases in which caregivers self-report the children’s medical diagnoses, as they may omit diagnoses or report conditions that have not yet been confirmed. Another aspect of this type of bias pertains to the scale used to measure food neophobia in this study, which does not directly assess children’s reactions but instead takes an indirect approach to eating behavior, relying on caregivers’ memories [52]. However, we believe that since most of these caregivers are mothers, they comprehensively understand the children regarding their habits, common reactions, aversions, and food preferences. We emphasize that the instrument used in this study was previously validated, and the scores of the FN assessment scale demonstrate strong psychometric properties [8].

Finally, another aspect to consider is the lack of analysis (or the failure to address it as a focal point) of potential confounding variables, such as family food consumption and the grouping of all neurodivergent individuals. Factors such as sensory sensitivity [50] and social deficits [49] may influence FN, depending on the condition. However, although these variables may influence or even increase the prevalence of food neophobia in certain conditions or contexts, the focus was on comparing food neophobia among neurodivergent individuals in general. This approach did not allow for the stratification of the analysis by specific variables or health conditions. More research is needed to explore and further investigate factors that influence and correlate with FN in neurodivergent individuals, including other strategies to complement, reduce, or eliminate this bias.

To help stabilize or minimize FN, healthcare professionals who care for these children should educate caregivers on the importance of multidisciplinary monitoring. These professionals, such as psychologists, nutritionists, and speech therapists, should evaluate the children and, when necessary, recommend feeding therapy, sensory integration therapy to address sensitivity, cooking workshops, nutritional supplementation, and psychotherapy. Professionals should continually monitor the child’s progress and propose adjustments to interventions, regardless of whether the child is neurotypical or neuroatypical.

Several factors influencing FN are related to parents and caregivers. Health professionals should be vigilant for signs of FN and provide support and guidance to parents to help them effectively manage this issue. Multidisciplinary work involving food and nutrition education, along with the engagement of parents and caregivers, can help make children’s eating habits less challenging and may even reduce FN levels.

## 5. Conclusions

Based on the comparison proposed between the groups in this study, neurodivergent Brazilian children exhibited greater FN compared to neurotypical children, and dietary restrictions in neurodivergent children had no significant influence on the prevalence or severity of FN. Additionally, dietary restrictions in neurodivergent children did not increase or decrease FN.

Regardless of whether a child is neurotypical or neurodivergent, with or without dietary restrictions, caregivers and health professionals must encourage and recommend sensory integration therapy to address sensitivity, cooking workshops, or other food and nutrition education strategies, psychotherapy, and nutritional supplementation. Furthermore, providing support, monitoring, and following up with children and their families should be integral to the multidisciplinary healthcare team’s work. Longitudinal studies and direct observation of behaviors may further elucidate the findings.

## Figures and Tables

**Table 2:** Distribution of the sample according to neophobia classification (n = 2387).

	Food Neophobia		
	Low n (%)	Moderate n (%)	High n (%)
Domain of neophobia in general * (FNgen)	516 (21.6%)	672 (28.2%)	1199 (50.2%)
Domain of neophobia for fruits * (FNfru)	760 (31.8%)	698 (29.2%)	929 (38.9%)
Domain of neophobia for vegetables * (FNveg)	535 (22.4%)	655 (27.4%)	1197 (50.1%)
TOTAL INSTRUMENT SCORE ** (BCFNeoTot)	575 (24.1%)	756 (31.7%)	1056 (44.2%)

* Domain score cutoff points: low (up to 13 points); moderate (14 to 21 points); high (22 points or more). ** Total score cutoff points: low (up to 40 points); moderate (41 to 65 points); high (66 points or more).

**Table 3:** Food neophobia score and classification distribution by sex and age group (n = 2387).

	Gender			Age		
	Girls (n = 1009)	Boys (n = 1378)	*p*	4–7 y (n = 1430)	8–11 y (n = 957)	*p*
General neophobia (FNgen)						
Score; Mean ± SD	19.22 ± 8.69	21.93 ± 8.68	<0.001 *	20.67 ± 8.67	20.95 ± 8.95	0.452 *
Distribution; n (%)						
Low (up to 13)	262 (26.0%)	254 (18.4%)		307 (21.5%)	209 (21.8%)	
Moderate (14 to 21)	324 (32.1%)	348 (25.3%)	<0.001 **	416 (29.1%)	256 (26.8%)	0.539 **
High (22 or more)	423 (41.9%)	776 (56.3%)		707 (49.4%)	492 (51.4%)	
Fruit neophobia (FNfru)						
Score; Mean ± SD	16.28 ± 8.45	19.10 ± 8.68	<0.001 *	17.85 ±8.67	18.00 ± 8.74	0.675 *
Distribution; n (%)						
Low (up to 13)	386 (38.3%)	374 (27.1%)		459 (32.1%)	301 (31.5%)	
Moderate (14 to 21)	321 (31.8%)	377 (27.4%)	<0.001 **	419 (29.3%)	279 (29.2%)	0.680 **
High (22 or more)	302 (29.9%)	627 (45.5%)		552 (38.6%)	377 (39.4%)	
Vegetable Neophobia (FNveg)						
Score; Mean ± SD	18.95 ± 8.27	21.32 ± 8.30	<0.001 *	20.30 ±8.38	20.35 ± 8.36	0.877 *
Distribution; n (%)						
Low (up to 13)	273 (27.1%)	262 (19.0%)		317 (22.2%)	218 (22.8%)	
Moderate (14 to 21)	302 (29.9%)	353 (25.6%)	<0.001 **	399 (27.9%)	256 (26.8%)	0.959 **
High (22 or more)	434 (43.0%)	763 (55.4%)		714 (49.9%)	483 (50.5%)	
TOTAL (BCFNeoTot)						
Score; Mean ± SD	54.45 ± 23.77	62.35 ± 24.11	<0.001 *	58.82 ±24.2	59.30 ± 24.39	0.634 *
Distribution; n (%)						
Low (up to 40)	281 (27.8%)	294 (21.3%)		353 (24.7%)	222 (23.2%)	
Moderate (41 to 65)	371 (36.8%)	385 (27.9%)	<0.001 **	454 (31.7%)	302 (31.6%)	0.349 **
High (66 or more)	357 (35.4%)	699 (50.7%)		623 (43.6%)	433 (45.2%)	

* Independent *t*-test; ** Mann–Whitney test.

**Table 4:** Food neophobia score and classification distribution considering neurodiversity and presence or absence of dietary restrictions (n = 2387).

	G1 (n = 767)	G2 (n = 133)	G3 (n = 126)	G4 (n = 1361)	*p*
General neophobia (FNgen)					
Score; Mean ± SD	23.86 ± 8.21 ^b^	23.38 ± 8.38 ^b^	18.16 ± 9.37 ^a^	19.03 ± 8.53 ^a^	<0.001 *
Distribution; n (%)					
Low (up to 13)	100 (13.0%)	15 (11.3%)	42 (33.3%)	359 (26.4%)	
Moderate (14 to 21)	149 (19.4%) ^b^	36 (27.1%) ^b^	31 (24.6%) ^a^	456 (33.5%) ^a^	<0.001 **
High (22 or more)	518 (67.5%)	82 (61.7%)	53 (42.1%)	546 (40.1%)	
Fruit neophobia (FNfru)					
Score; Mean ± SD	21.32 ± 8.15 ^b^	21.37 ± 8.24 ^a^	15.03 ± 8.40 ^a^	15.92 ± 8.35 ^a^	<0.001 *
Distribution; n (%)					
Low (up to 13)	138 (18.0%)	21 (15.8%)	55 (43.7%)	546 (40.1%)	
Moderate (14 to 21)	199 (25.9%) ^b^	43 (32.3%) ^b^	40 (31.7%) ^a^	416 (30.6%) ^a^	<0.001 **
High (22 or more)	430 (56.1%)	69 (51.9%)	31 (24.6%)	399 (29.3%)	
Vegetable Neophobia (FNveg)					
Score; Mean ± SD	22.89 ± 8.17 ^b^	22.39 ± 8.75 ^b^	17.75 ± 8.33 ^a^	18.91 ± 8.04 ^a^	<0.001 *
Distribution; n (%)					
Low (up to 13)	108 (14.1%)	24 (18.0%)	45 (35.7%)	358 (26.3%)	
Moderate (14 to 21)	168 (21.9%) ^b^	25 (18.8%) ^b^	35 (27.8%) ^a^	427 (31.4%) ^a^	<0.001 **
High (22 or more)	491 (64.0%)	84 (63.2%)	46 (36.5%)	576 (42.3%)	
TOTAL (BCFNeoTot)					
Score; Mean ± SD	68.07 ± 22.90 ^b^	67.14 ± 23.47 ^b^	50.94 ± 24.98 ^a^	53.86 ± 23.28 ^a^	<0.001 *
Distribution; n (%)					
Low (up to 40)	107 (14.0%)	17 (12.8%)	50 (39.7%)	401 (29.5%)	
Moderate (41 to 65)	178 (23.2%) ^b^	33 (24.8%) ^b^	39 (31.0%) ^a^	506 (37.2%) ^a^	<0.001 **
High (66 or more)	482 (62.8%)	83 (62.4%)	37 (29.4%)	454 (33.4%)	

* One-way ANOVA with Tukey’s post hoc test; ** Kruskal–Wallis with Dunn’s post hoc test. For the post hoc tests, the same letters do not differ significantly. G1: neurodivergent children without dietary restrictions; G2: neurodivergent children with dietary restrictions; G3: neurotypical children with dietary restrictions; and G4: neurotypical children without dietary restrictions.

## Data Availability

The original contributions presented in this study are included in the article/Supplementary Material. Further inquiries can be directed to the corresponding author.

## Supplementary Materials

The following supporting information can be downloaded at https://www.mdpi.com/article/10.3390/nu17081327/s1, Table S1: Characterization of caregivers and their children (n = 2387). Table S2: Characterization of children’s medical conditions and diagnoses (n = 2387).
