# Supplementary material for: Food Neophobia in Brazilian Children: A Nationwide Cross-Sectional Study Comparing Neurodivergent and Neurotypical Children with and Without Dietary Restrictions
Source: Nutrients. 2025 Apr 11;17(8):1327. doi: 10.3390/nu17081327 (PMC12030263; doi:10.3390/nu17081327)
Supplement: Supplementary file 1 [file nutrients-17-01327-s001.zip › nutrients-3564446-supplementary.pdf]

Table S1. Characterization of caregivers and their children (n=2387).

	Categories	Sample	
		**n**	**%**
**Caregivers Sex**	Female	2228	93.3
	Male	159	6.7
**Caregivers' degree of kinship**	Mothers	2108	88.3
	Fathers	143	6.0
	Stepmother or stepfather	10	0.5
	Grandparents	42	1.7
	Sisters or brothers	26	1.0
	Uncles or aunts	36	1.5
	Other degree of kinship	11	0.5
	Unrelated	11	0.5
**Caregiver Marital status**	Single	290	12.1
	Married/ stable union	1884	78.9
	Separate/ divorced	193	8.1
	Widower	20	0.8
**Educational Level of Caregivers**	From 1st to 4th grade of elementary school (former primary)	9	0.4
	From 5th to 8th grade of elementary school (former gym)	46	1.9
	High School (2nd degree) incomplete	69	2.9
	Complete high school	335	14.0
	Higher Education Incomplete	244	10.2
	Higher Education Complete	497	20.8
	Postgraduate	758	31.8
	Master's degree	218	9.1
	Doctorate degree	170	7.1
	Postdoctoral	41	1.7
**Housing area**	Urban area	2299	96.3
	Rural area	87	3.6
	Indigenous area	1	0.1
**Monthly family income**	No income	58	2.4
	Up to 1 MW	248	10.4
	Up to 2 MW	266	11.1
	Up to 3 MW	214	9.0
	Up to 4 MW	172	7.2
	Up to 5 MW	142	5.9
	Up to 6 MW	113	4.7
	Up to 7 MW	90	3.8
	Up to 8 MW	79	3.3
	Up to 9 MW	96	4.0
	Between 10 and 12 MW	268	11.2

Between 13 and 15 MW	139	5.8
Above 15 MW	343	14.4
Not informed	159	6.7

MW: Minimum wage. Brazil MW in 2024: R$ 1.412,00, about USD $220 (Conversion rate of USD 1.00 to R$6.35, in december 2024).

Table S2. Characterization of children's medical conditions and diagnoses (n=2387).

**Medical condition and/or diagnoses**	**Sample**	
	**n**	**%**
Down syndrome	197	8.3
Autism spectrum disorder	478	20.0
Hearing impairment	1	0.0
Sensory processing disorder	6	0.3
Eating disorders[a]	6	0.3
Thyroid disease[b]	3	0.1
Cerebral palsy	1	0.0
Attention Deficit Hyperactivity Disorder	15	0.6
Hyperactivity	1	0.0
Intellectual disability	1	0.0
Irlen syndrome	2	0.1
Anxiety	3	0.1
High abilities	3	0.1
Asthma	16	0.7
Fragile X syndrome	1	0.0
Food allergy[c]	49	2.1
Food intolerance[d]	47	2.0
Gastroesophageal reflux	3	0.1
Atopic dermatitis	4	0.2
Glucose-6-phosphate dehydrogenase (G6PD) deficiency	2	0.1
Inflammatory bowel disease	1	0.0
Diabetes	1	0.0
No diagnosis	1294	54.2
Agitated, nervous, dye allergies and aggressive	1	0.0
Precocious puberty	4	0.2
Allergic rhinitis	6	0.3
Sickle cell anemia	1	0.0
Asthmatic or allergic bronchitis	4	0.2
Amygdalate -Tonsillitis	1	0.0
Muscular dystrophy	1	0.0
Total atrioventricular block	1	0.0
Contact dermatitis	1	0.0
Idiopathic or juvenile rheumatoid arthritis	2	0.1
Speech difficulties	1	0.0
Thaleseemia	2	0.1
Convergence syndrome	1	0.0
Down syndrome and Autism spectrum disorder	5	0.2
Down syndrome and heart problems	3	0.1
Down syndrome and hearing impairment	1	0.0
Down syndrome and Sensory processing disorder	1	0.0

Down syndrome and Eating disorders[a]	1	0.0
Down syndrome and Thyroid disease[b]	2	0.1
Down syndrome and brain injury	1	0.0
Down syndrome and vision problems[e]	1	0.0
Down syndrome and Hirschsprung's disease	1	0.0
Down syndrome and apraxia of speech	1	0.0
Down syndrome and Attention Deficit Hyperactivity Disorder	1	0.0
Down syndrome and Food allergy[c]	12	0.5
Down syndrome and Food intolerance[d]	21	0.9
Down syndrome and dye allergies	1	0.0
Down syndrome and claes-jensen syndrome	1	0.0
Down syndrome, Sensory processing disorder and Eating disorders[a]	1	0.0
Autism spectrum disorder and hearing impairment	1	0.0
Autism spectrum disorder and Sensory processing disorder	2	0.1
Autism spectrum disorder and Eating disorders[a]	8	0.3
Autism spectrum disorder and Cerebral palsy	3	0.1
Autism spectrum disorder and hydrocephalus	1	0.0
Autism spectrum disorder and Attention Deficit Hyperactivity Disorder	9	0.4
Autism spectrum disorder and epilepsy	3	0.1
Autism spectrum disorder and selective mutism	1	0.0
Autism spectrum disorder and oppositional defiant disorder	1	0.0
Autism spectrum disorder and Intellectual disability	2	0.1
Autism spectrum disorder and Goldenhar Syndrome	1	0.0
Autism spectrum disorder and Food allergy[c]	19	0.8
Autism spectrum disorder and Food intolerance[d]	37	1.6
Autism spectrum disorder and glucose-6-phosphate dehydrogenase (G6PD) deficiency	1	0.0
Heart problems and high cholesterol	1	0.0
Attention Deficit Hyperactivity Disorder and oppositional defiant disorder	1	0.0
Attention Deficit Hyperactivity Disorder and Anxiety	2	0.1
Attention Deficit Hyperactivity Disorder and Asthma	1	0.0
Attention Deficit Hyperactivity Disorder and Food allergy[c]	2	0.1
Epilepsy and Food allergy[c]	1	0.0
Asthma and allergic rhinitis	1	0.0
Food allergy[c] and Food intolerance[d]	15	0.6
Food allergy[c] and Gluten-related disorders	1	0.0
Food allergy[c] and Atopic dermatitis	4	0.2
Food allergy[c] and Glucose-6-phosphate dehydrogenase (G6PD) deficiency	1	0.0
Food allergy[c] and Potocki-Lupski syndrome	1	0.0
Food allergy[c] and prematurity	1	0.0
Food intolerance[d] and Celiac disease	1	0.0
Food intolerance[d] and language disorder	1	0.0
Down syndrome, Autism spectrum disorder and microcephaly	1	0.0
Down syndrome, Autism spectrum disorder and Food intolerance[d]	1	0.0
Down syndrome, Heart problems and apraxia of speech	1	0.0
Down syndrome, Cerebral palsy and Food allergy[c]	1	0.0
Down syndrome, Food allergy[c] and Food intolerance[d]	8	0.3
Down syndrome, Food allergy[c] and Celiac disease	1	0.0
Autism spectrum disorder, Sensory processing disorder and Food intolerance[d]	2	0.1

Autism spectrum disorder, Eating disorders[a] and Oppositional defiant disorder	1	0.0
Autism spectrum disorder, Attention Deficit Hyperactivity Disorder and epilepsy	1	0.0
Autism spectrum disorder, Attention Deficit Hyperactivity Disorder and Oppositional defiant disorder	2	0.1
Autism spectrum disorder, Attention Deficit Hyperactivity Disorder and Food allergy[c]	1	0.0
Autism spectrum disorder, epilepsy, and periventricular leukomalacia	1	0.0
Autism spectrum disorder, epilepsy, and Food intolerance[d]	1	0.0
Autism spectrum disorder, Hyperactivity, and Intellectual disability	1	0.0
Autism spectrum disorder, Hyperactivity, and Food allergy[c]	1	0.0
Autism spectrum disorder, Food allergy[c,] and Food intolerance[d]	10	0.4
Autism spectrum disorder, Food intolerance and Dubowit syndrome	1	0.0
Eating disorders[a], Food allergy[c] and Food intolerances	1	0.0
High abilities, Food intolerance[d,] and Gastroesophageal reflux	1	0.0
Asthma, Food intolerance[d,] and Atopic dermatitis	1	0.0
Food allergy[c] , Food intolerance[d,] and dermatitis herpetiformis	1	0.0
Food allergy[c], Celiac disease, and eosinophilic esophagitis	1	0.0
Down syndrome, Autism spectrum disorder, Food allergy[c,] and Food intolerance[d]	1	0.0
Autism spectrum disorder, Eating disorders[a], Food allergy[c,] and Food intolerance[d]	1	0.0
Autism spectrum disorder, Attention Deficit Hyperactivity Disorder, epilepsy and Oppositional defiant disorder	1	0.0
Autism spectrum disorder, Food allergy[c] , Food intolerance[d] and megacolon	1	0.0
Autism spectrum disorder, Food allergy[c] , Food intolerance[d] and Gluten-related disorders	1	0.0
Autism spectrum disorder, Food intolerance[d], Atopic dermatitis and neuromuscular syndrome	1	0.0
Down syndrome, Food intolerance[d] , Celiac disease, colitis and alopecia	1	0.0
Autism spectrum disorder, Food intolerance[d], Sensory processing disorder, Attention Deficit Hyperactivity Disorder and Oppositional defiant disorder	1	0.0
Autism spectrum disorder, Eating disorders[a], Intellectual disability, Food allergy[c] and Food intolerance[d]	1	0.0

[a]Anorexia/bulimia/pediatric eating disorder; [b]hypothyroidism, hyperthyroidism or Hashimoto's thyroiditis; [c] food allergies such as cow's milk, peanuts, wheat etc.; [d] food intolerances such as lactose, FODMAP, etc.; [e] low vision or blindness.
